# Future Perspectives in Spinal Cord Repair: Brain as Saviour? TSCI with Concurrent TBI: Pathophysiological Interaction and Impact on MSC Treatment

**DOI:** 10.3390/cells10112955

**Published:** 2021-10-30

**Authors:** Paul Köhli, Ellen Otto, Denise Jahn, Marie-Jacqueline Reisener, Jessika Appelt, Adibeh Rahmani, Nima Taheri, Johannes Keller, Matthias Pumberger, Serafeim Tsitsilonis

**Affiliations:** 1 Charité–Universitätsmedizin Berlin, Corporate Member of Freie Universität Berlin and Humboldt-Universität zu Berlin, Center for Musculoskeletal Surgery, Augustenburger Platz 1, 13353 Berlin, Germany; paul.koehli@charite.de (P.K.); ellen.otto@charite.de (E.O.); denise.jahn@charite.de (D.J.); marie-jacque.reisener@charite.de (M.-J.R.); jessika.appelt@charite.de (J.A.); adibeh.rahmani@charite.de (A.R.); nima.taheri@charite.de (N.T.); 2 Berlin Institute of Health at Charité–Universitätsmedizin Berlin, Julius Wolff Institute, Augustenburger Platz 1, 13353 Berlin, Germany; 3 Berlin Institute of Health at Charité–Universitätsmedizin Berlin, Charitéplatz 1, 10117 Berlin, Germany; j.keller@uke.de; 4 University Hospital Hamburg-Eppendorf, Department of Trauma Surgery and Orthopaedics, Martinistraße 52, 20246 Hamburg, Germany

**Keywords:** traumatic spinal cord injury, TSCI, traumatic brain injury, TBI, mesenchymal stem cells, MSC

## Abstract

Traumatic spinal cord injury (TSCI), commonly caused by high energy trauma in young active patients, is frequently accompanied by traumatic brain injury (TBI). Although combined trauma results in inferior clinical outcomes and a higher mortality rate, the understanding of the pathophysiological interaction of co-occurring TSCI and TBI remains limited. This review provides a detailed overview of the local and systemic alterations due to TSCI and TBI, which severely affect the autonomic and sensory nervous system, immune response, the blood–brain and spinal cord barrier, local perfusion, endocrine homeostasis, posttraumatic metabolism, and circadian rhythm. Because currently developed mesenchymal stem cell (MSC)-based therapeutic strategies for TSCI provide only mild benefit, this review raises awareness of the impact of TSCI–TBI interaction on TSCI pathophysiology and MSC treatment. Therefore, we propose that unravelling the underlying pathophysiology of TSCI with concomitant TBI will reveal promising pharmacological targets and therapeutic strategies for regenerative therapies, further improving MSC therapy.

## 1. Introduction

Traumatic spinal cord injury (TSCI), commonly caused by high energy trauma such as traffic accidents and falls [1,2], results in temporary to permanent loss of perception of tactile sensation, neuromuscular function, autonomous dysregulation and even death. TSCI affects 13.1 to 52.2 million often young active patients per year, impairing predominantly the cervical spine (41.6–75%) but also the thoracic (16–41%) and lumbar spine (9–17.6%) [3]. The consequences of TSCI vary depending on the localisation of the lesion, ranging from locked-in syndrome with the need for continuous external ventilation (high cervical spine) to cauda syndrome with loss of bladder and rectal control (lumbar spine) [4]. TSCI is frequently accompanied by additional injuries [5,6,7,8], in particular traumatic brain injury (TBI) in up to 60% of cases, which not only results in reduced clinical outcomes but also in higher mortality [5,6,7,8]. Overall, TSCI represents a major long-term physical, psychological and socioeconomic impact on patients and their families as well as the entire health care system [9,10,11,12,13].

To date, the standard of care for TSCI and TBI consists of preclinical immobilisation, a guideline-compliant emergency diagnostic process with subsequent surgical decompression and spinal stabilisation, followed by postoperative intensive care and early physical rehabilitation [14]. The evidence for additional pharmacological interventions in acute settings, such as glucocorticoids, remains controversial [15,16]. Even with subsequent long-term rehabilitation and medical and psychological care, the percentage of long-lasting disabilities remains high, raising the essential need for new regenerative therapeutic options such as stem cell-based therapies. Such therapies in TSCI aim to promote neural axonal regeneration and functional restoration by limiting the secondary injury, while optimising healing cascades through modulation of the local microenvironment and inflammatory process through targeted differentiation of the added cells [17]. 

Recent preclinical and clinical phase I/II TSCI studies focusing on mesenchymal stem cell (MSC) therapies showed an acceptable risk profile, with meta-analysis concluding minor improvement in TSCI outcome that remains far from total recovery [18,19]. As the complexity of the interactions of neural injury and repair with posttraumatic whole-body pathophysiology is not yet fully elucidated [20,21], a deeper understanding of these interactions could prove crucial to optimising the local and systemic regenerative effects of MSC-based therapies.

The comprehension of the pathophysiological interaction between TSCI and TBI on a molecular level and its impact on the whole organism remains limited. In this review, we provide a detailed overview of the local and systemic alterations resulting from TSCI and TBI which severely affect the autonomic and peripheral nervous system, inflammatory response, local perfusion, endocrine homeostasis and circadian rhythm. Based on this mechanistic understanding, we aim to raise awareness for the currently neglected impact of these effects on MSC-based regenerative therapies following TSCI.

## 2. Methods

The MEDLINE database was searched, applying the following keywords: spinal cord injury, SCI, Traumatic spinal cord injury, TSCI, traumatic brain injury, TBI, bone fracture, fracture, trauma, circadian, metabolism, endocrine, hormone, autonomous nervous system, sympathet*, parasympathet*, adrenerg*, immune, perfusion, regenerative therapies, and mesenchymal stem cells (MSC), in different combinations. The MEDLINE database research was carried out between 1 April and 1 August 2021. For identification of additional studies, the bibliographies of identified papers were analysed. For the brief overview of registered clinical trials regarding cell therapy in TBI and TSCI, we performed a systematic search on the clinicaltrials.gov register on the 1 of October with respect to PRISMA statements [22,23]. Therefore, traumatic brain injury, respective spinal cord injuries and ((stem-cells) OR (stem cells) OR cells OR cell) was used as a search term, and studies not using cell therapies or not aiming for TBI or TSCI treatment were excluded. The remaining studies were analysed. The publication of results was identified based on a search of the NCT in the MEDLINE-Database, and on google-scholar if not successful based on the search of the registered PI in both data bases. If neither of the two search strategies resulted in any hits for the registered clinical study, the trial was rated as not published.

## 3. Interaction of TSCI and TBI

### 3.1. A Brief Pathophysiology of Isolated TSCI and TBI

**Figure 1:**
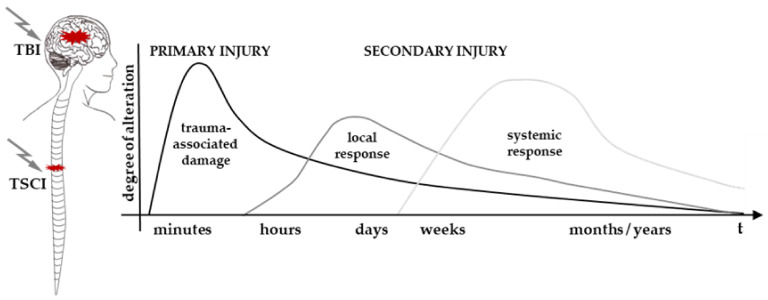
Pathophysiological course of events following acute damage of the central nervous system: TSCI and TBI. Once the primary injury occured, which represents the initial mechanical insult, the secondary injury is induced within minutes as a local response. Through the additional compromised blood–brain and blood–spinal cord barrier and further mechanisms discussed in this review, the local response evolves into a systemic one, followed by regenerative processes. Abscissa axis = time; ordinate axis = degree of relative alteration. Graphic adapted from [26]. Abbreviations: TBI = traumatic brain injury, TSCI = traumatic spinal cord injury.

The neurotraumatic injuries TSCI and TBI are caused by an external physical insult which results in a force-dependent temporary to permanent functional alteration. The highly complex pathophysiology of both TSCI and TBI is divided into primary injuries, induced by the initial energy transfer and deformation, and secondary injuries, referring to the subsequent biochemical and cellular cascade at the local and systemic levels in response to the primary event; these injuries can range from acute to chronic (Figure 1) [1,24,25].

In TSCI, the primary injury causes the displacement of vertebral bone and disco-ligamentous structures resulting in transient or permanent compression, contusion (closed injury), distraction or laceration (open injury) with transection of the spinal cord and its vasculature, thus leading to ischemia [1,27,28]. Additionally, descending sympathetic nerve fibres are commonly compromised, further impairing autoregulatory processes. This can result in neurogenic shock by loss of sympathetic tone with consecutively decreased peripheral vascular resistance and reduced cardiac output, resulting in systemic hypotension and hypoperfusion [29,30]. The secondary injury is divided into acute (0–48 h), subacute (2–14 days), intermediate (14 days–6 months) and chronic phases (>6 months). During the acute and subacute phases, the synergistic effects of traumatic cell damage, ischemia, oedema and inflammation lead to cellular dysfunction, pro-apoptotic signalling and cell death [31], resulting in the breakdown of the blood–spinal cord barrier [30,32]. Regenerative mechanisms facilitate local and systemic innate immune responses, while also antagonizing the traumatic transitory ischemia [33,34]. Therefore, angiogenesis is one of the first regenerative response mechanisms upregulated after TSCI [33,35]. Although new vessels often lack astrocyte- [36] or pericyte-association [37], endogenous mechanisms target neuronal functional regeneration through axon growth stimulation, leading to collaterals originating from undamaged axons [38,39]. Nevertheless, even though Schwann cells and oligodendrocytes migrate to the injury epicentre and re-myelinate structures, the original function remains impaired, as cystic cavities and glial scarring form a barrier surrounding severely damaged tissue [39,40,41,42,43,44,45].

TBI is commonly classified by injury severity into mild, moderate and severe based on the level of consciousness [46] as well as by neuropathological features, dividing brain damage into focal (through direct impact) and diffuse (through acceleration/deceleration) injury [47,48]. While diffuse injury causes oedema, concussion and diffuse axonal damage, focal brain damage results in contusion (closed injury), laceration (open injury) or intracranial haemorrhage. Similar to TSCI, the primary injury represents the initial mechanical damage of the meninges, neurons and vasculature, while subsequent secondary injury refers to the delayed non-mechanical damage [24,49,50,51]. Therefore, secondary injury arises from damaged parenchyma and vasculature causing ischemic injury, ionic imbalance with depolarisation and excitotoxicity, free-radical generation and oxidative stress, which results in the activation of glial cells such as astrocytes and microglia [25]. As a result, the secondary injury induces cerebral hypoxia, oedema formation and intracranial hypertension, disruption of the neuronal networks [52], and impairment of the blood–brain barrier [53] with local and systemic inflammation [54]. Although the activation of microglia and astrocytes is crucial for regeneration through the rapid clearance of debris such as haematoma as well as for partial restoration of the blood–brain barrier and production of neurotrophic factors [25], they also release cytokines and chemokines that recruit circulating neutrophils and macrophages to the injured area, thus facilitating the inflammatory response [55]; this can prove to be a ‘double-edged sword’ [56]. In any case, neuro-inflammation represents the foundation for posttraumatic recovery of the central nervous system (CNS) [55], leading to gliosis, pericyte activation, glial scarring and extracellular matrix as well as angiogenesis, limited axonal regeneration, neurite sprouting, and neuro- and oligodendrogenesis [57]. Unfortunately, TSCI and TBI often result in chronic neuro-inflammation, astrocyte hyperactivation, pericyte persistence, extracellular matrix deposition and glial scar formation, acting as a physical and biochemical barrier to regeneration [55,57,58].

### 3.2. General Interaction of TSCI and TBI

As TSCI and TBI represent major traumata with profound systemic effects, they are followed by metabolic and immune alterations potentially resulting in energy wasting, systemic inflammatory response syndrome (SIRS) and critical illness with the need for intensive care, which poses a major challenge to regeneration and healing processes (Section 3.2, Section 3.3, Section 3.4, Section 3.5, Section 3.6, Section 3.7 and Section 3.8). While isolated TSCI and TBI were reported to result in cognitive impairment [8,59,60], TSCI in polytrauma patients [61] as well as TSCI with concomitant TBI showed additive effects with further reduced cognitive and neuromotor outcomes [8,59,60,61]. Therefore, long-term mood disorders, polypharmacy, post-intensive care unit syndrome, cortical reorganisation and neuro-inflammation are recognised as additional therapeutic challenges of TSCI with concurrent TBI [8].

Polytrauma patients often present with additional high-energy musculoskeletal injuries that further deteriorate the clinical outcome in patients with TSCI [62]. Both TSCI and TBI are further reported to reduce bone quality, potentially as a result of afferent signalling from brain and spinal cord to bone and muscle [63,64,65,66]. Opposite to the afferent interaction, concomitant fractures were reported to negatively affect cerebral oedema formation and delay cognitive recovery following TBI [67,68]. Therefore, efferent effects of bone and muscle on the brain and their therapeutic potential represent a contemporary issue [69,70]. While concomitant bone fractures showed a negative impact on the clinical outcome of TBI patients, efferent effects from bone and muscle on TSCI have not been studied yet.

In contrast to the negative effect on bone quality, TSCI and TBI were both identified to cause heterotopic ossifications (HO) [65] and improved healing of concomitant long-bone fractures in humans [71,72]. Therefore, experimental models have been developed in order to investigate the underlying mechanisms [64,68,73,74,75]. Clinical observations of spinal cord injury report different risk factors for HO, including complete injuries, spasticity and pneumonia [76]. To date, the early detection and management of HO after traumatic neuronal injury remains a clinical challenge. Although HO following TSCI and TBI is suggested to share the same pathophysiology, knowledge of the underlying mechanisms remains limited [77,78]. A detailed understanding of the molecular processes during HO formation provides great future potential for new therapeutic targets to improve bone healing as well as to inhibit prevalent HO.

Surprisingly, only one preclinical model has been published to date, combining unilateral cervical TSCI and unilateral (ipsi- vs. contralateral) TBI in rats [79]. In the model, TBI contralateral to TSCI further decreased motoric function ipsilateral to TSCI in the frontal limb, while ipsilateral TBI resulted in slightly improved function compared to isolated TSCI at six weeks post-injury. This outcome was considered to result from a balance of motor innervation by ipsilateral TBI or enhanced neuroplastic coping mechanisms such as central neuroplastic adaption, which was discussed in chronic cervical myelopathy [80]. Finally, this animal model suggests that during the development of treatment plans for patients with multiple injuries, the balanced activity and potential inhibitory effects of residual functional central systems must be taken into consideration.

**Figure 2:**
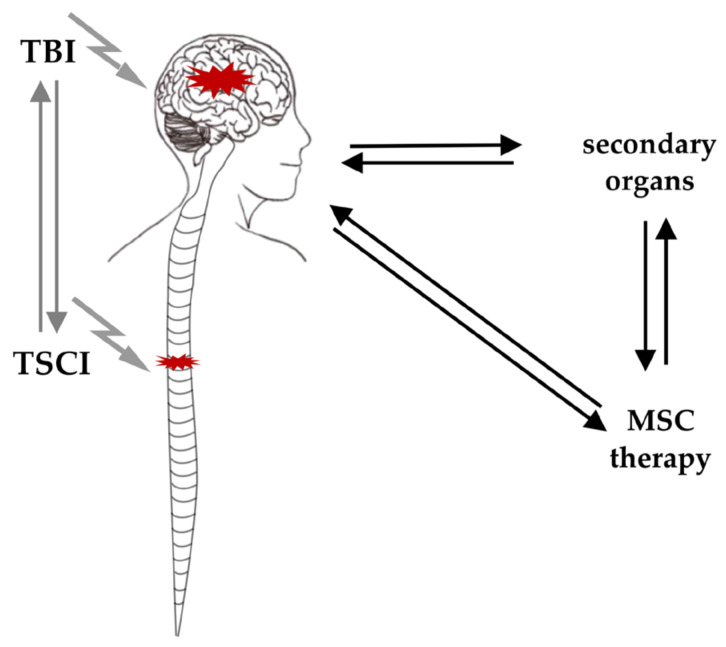
TSCI and TBI interaction occurs directly or indirectly via secondary organs. TBI and TSCI can influence each other directly via neurogenic interactions or indirectly via their impact on secondary organs, tissues and signalling cascades. MSC therapy in this context may influence TSCI or TBI directly or by their impact on these secondary alterations. In concomitant TSCI and TBI the role of MSC treatment is unclear, and whether the established options for one injury affect the other also remains unclear. Abbreviations: MSC = mesenchymal stem cell, TBI = traumatic brain injury, TSCI = traumatic spinal cord injury.

In summary, the negative effects of TSCI on cognitive function in patients suffering from TBI as well as the impact of TBI on the sensorimotor outcome in patients with TSCI suggest a bidirectional interaction of both traumatic injuries. This interaction might occur directly or indirectly via secondary organs (Figure 2). This is further supported by the clinical observation of enhanced bone formation following TSCI and TBI, which employ local regenerative mechanisms involving MSCs [81,82] and underline the impact on MSC-mediated healing cascades. Furthermore, concomitant injuries, diseases and complications can influence the course of healing [83]. However, the current understanding of TSCI-TBI crosstalk and its effect on MSC treatment remains limited [84].

### 3.3. Autonomic Dysregulation after TSCI and TBI

**Figure 3:**
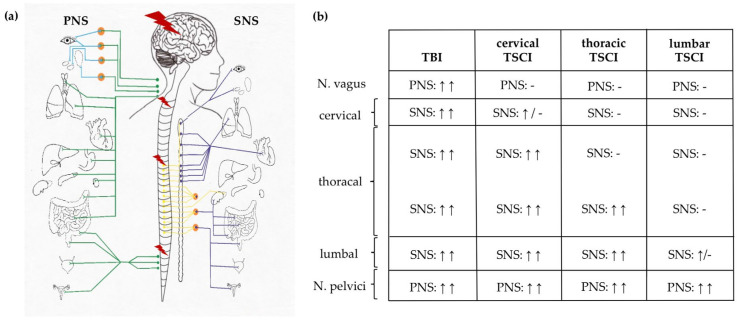
Differential effects of TSCI and TBI on the autonomic nervous system (ANS) depending on the trauma level. (**a**) The parasympathetic innervation of thoracic and upper abdominal organs originates from the cranial nerve N. vagus, while the innervation of the lower abdominal and pelvic organs (Cannon’s point) originates from the spinal cord segments S2-S4 (Nn. splanchnici pelvicii). The sympathetic innervation via the sympathetic trunk originates from the spinal cord segments T1-L3. (**b**) While TBI results in general dysregulation (↑↑) of PNS and SNS, TSCI commonly spares the N. vagus (except for high cervical trauma), while its effects on the SNS are dependent on the localisation of trauma. Graphics adapted from [4,85]. Abbreviations: N. = Nervous, PNS = parasympathetic nervous system, SNS = sympathetic nervous system, TBI = traumatic brain injury, TSCI = traumatic spinal cord injury.

As part of the peripheral nervous system, the autonomous nervous system (ANS) consists of the parasympathetic nervous system (PNS), the sympathetic nervous system (SNS) and the enteric nervous system. The PNS originates from the brain stem with the cranial nerves and Nervus vagus, as well as from the distal spinal cord as Nn. splanchnici pelvicii (segments S2-S4). N. vagus directly controls thoracic and abdominal organs, and is predominantly associated with enhanced digestive function and reduced state of general activation of the body, such as lower blood pressure and heart rate [85]. On an organ level, PNS effects are mediated by chemical synapses via acetylcholine transmission on muscarinergic acetylcholine receptors. While the parasympathetic role of N. vagus remains unquestioned, a possible sympathetic origin of Nn. splanchnici pelvicii was discovered and further discussed in recent literature [86,87]. The first neurons of the SNS originate from the lower cervical to lumbar spinal cord (segments T1-L3) and innervate the second neurons in the sympathetic trunk, which proceed parallel from the upper cervical to the lower sacral spine (Figure 3a) [85,88]. The end organ effect of the SNS is mediated by norepinephrine from sympathetic nerves and epinephrine from the adrenal glands, typically involved in ‘fight or flight’ reactions including acceleration of heart rate, blood pressure and reduction of digestive activity [85]. The enteric nervous system is the largest part of the ANS and consists of independent microcircuits allowing gastrointestinal coordination without active input from the central nervous system [89].

In TSCI, acute and chronic autonomic dysregulation represent a serious complication. In the acute event of trauma, the loss of central control of sympathetic and parasympathetic innervation below the injury potentially results in neurogenic shock, whereas long-term consequences such as neurogenic bladder and bowel dysfunction, reduced gastrointestinal motility, and sympathetic dysregulation causing pain syndromes are tremendous effects leading to poor quality of life. Depending on the segmental localisation of TSCI, differential effects on PNS and SNS can be expected (Figure 3b). While cervical TSCI results in general autonomy of the SNS and lower PNS, thoracic and lumbar TSCI cause partial or no dysfunction at all of the SNS, but loss of function of the lower PNS including autonomous bladder and bowel dysfunction [90]. Spinal fractures or surgical interventions can cause additional direct trauma to the sympathetic trunk or the N. vagus and the Nn. splanchnici pelvicii [91].

While general trauma results in an indirect rise of SNS tone, TBI can cause a direct rise of SNS tone [92,93]. Following TBI, enhanced autonomic activity of the PNS has been reported [94]. The rise in PNS tone of N. vagus was linked to posttraumatic immunosuppression with enhanced risk of posttraumatic pneumonia [94,95,96] and insufficient cardiovascular adaption [97,98]. Similar to TBI, posttraumatic dysregulation of the PNS following TSCI was linked to enhanced immunosuppression [99], cardiovascular deterioration [100], and neurogenic bowel disease with consequent malnutrition and dysregulation of microbiota [101]. Therefore, ANS dysregulation with additive effects on whole systems biology in TSCI patients with concomitant TBI certainly contributes to enhanced morbidity and mortality.

Along with ANS dysregulation after trauma, SNS and PNS are further involved in neural regeneration, and therefore in TSCI–TBI outcome and complications. Acute activation of the SNS following TSCI and TBI has been postulated to reduce immunoactivity, resulting in an enhanced risk for infections [92,102]. While the increased posttraumatic SNS activity impairs TSCI regeneration and outcome through reduced local perfusion, the beta adrenoreceptor antagonists and alpha-2 agonists positively influence the ischemic injury and reduce tissue inflammation of neural damage in vivo and in vitro [103,104]. However, the increased posttraumatic SNS activity further induces the browning of white adipose tissue and the activation of brown adipose tissue [105,106,107], previously linked to improved neural regeneration following TSCI [108] and TBI [109]. Further, norepinephrine has been linked to reduced MSC apoptosis [110], and adrenergic receptors are involved in MSC differentiation [111].

As TSCI and TBI both differentially affect the SNS, additive effects and systemic influences after combined trauma on the immune response, endocrine system, haemodynamics, energy metabolism and stem-cell differentiation are likely and warrant further studies.

### 3.4. Nociceptive Peptides

Nociceptive neuropeptides of the sensory nervous system play a crucial role in neuroinflammation and persistent pain syndromes secondary to spinal and peripheral nerve injury following TSCI, as well as in TBI-induced cerebral oedema [112,113,114]. In response to TBI, local and systemic alterations of substance P, calcitonin gene-related peptide (CGRP) and neuropeptide Y (NPY) have been monitored [114,115,116]. In TSCI, these peptides are involved in the development of chronic pain [113] and seem to play a role in neuroinflammation and healing after TSCI [117]. However, whether TSCI regenerative processes are altered by TBI via the sensory nervous system remains to be elucidated.

### 3.5. Immune Response and the Blood–Brain/Blood–Spinal Cord Barriers

**Figure 4:**
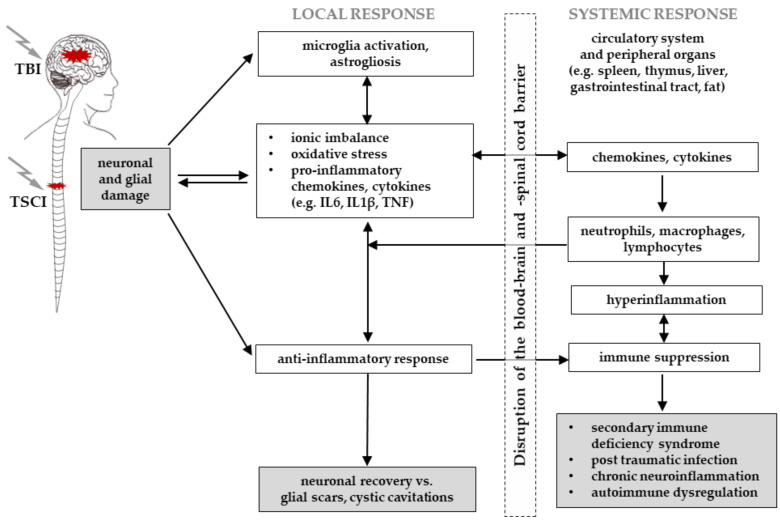
Proposed local and systemic immune response following injury to the central nervous system: TSCI and TBI. The initial primary injury causes neuronal and glial as well as meningeal and vascular damage, which activates the local innate immunity. In response, the level of pro- and anti-inflammatory molecules increases, which triggers peripheral immune cells to access the injury site through the disrupted blood–brain and blood–spinal cord barriers, resulting in a systemic immune response. While the balance of pro- and anti-inflammatory mediators induces repair processes aiming for neuronal recovery, potentially leaving glial scars and cystic cavitations, a dysfunctional response can result in systemic hyperinflammation with damage to peripheral organs and sepsis, chronic neuroinflammation, and autoimmunity, as well as systemic immune suppression and secondary immune deficiency syndrome. Graphic inspired by [123]. Abbreviations: IL = interleukin, TBI = traumatic brain injury, TNF = tumour necrosis factor, TSCI = traumatic spinal cord injury.

The immune response after central nervous system injury follows a ubiquitous and well-orchestrated cascade of inflammatory events [57,58]. At the time point of the primary injury, neuronal and glial damage, meningeal contusion or laceration, and disruption of the vasculature and descending/ascending signalling pathways occur [32,52]. These events activate the innate immune response of the central nervous system. Therefore, resident microglia and astrocytes start the clearance of damaged tissue, partially seal the barriers, and produce neurotrophic factors. In parallel, regenerating cytokines, chemokines, reactive oxygen species and excitatory neurotransmitters recruit and activate circulating neutrophils and macrophages to the injured area [25,57,58]. The subsequent release of these mediators triggers the secondary injury [25,118], thus resulting in direct epithelial damage, excitotoxicity and oxidative stress, ionic and therefore osmotic imbalance, oedema, increasing intracranial pressure with decrease of cerebral perfusion pressure, reduced cerebral blood flow, and hypoxia [25]. All these mechanisms further contribute to the progression of blood–brain barrier breakdown [119] following TBI and blood–spinal cord barrier breakdown [53] after TSCI. Depending on the pattern and the severity of the injury, the function of both barriers can be compromised simultaneously, further amplifying and elongating the progression of the secondary injury and neuroinflammation. Barrier dysfunction can be observed for weeks to months after TSCI [32,120], or even years following TBI [121], even in sites distant to the injury along the spinal cord axis [120]. Barrier breakdown allows the recruitment and pathophysiological interaction of the mediators and peripheral immune cells, such as monocytes, that differentiate into macrophages, neutrophils and lymphocytes, thus initiating a systemic immune response [25,54,122,123] (Figure 4).

Although the immune response after central nervous system injury follows a ubiquitous molecular and cellular cascade, TSCI and TBI were observed to show distinct neuroinflammatory reactions in terms of composition, spatiotemporal sequence and magnitude of response [42,124]. After TBI and TSCI, the level of glial activation and inflammatory response differs, with increased cytokine expression of inflammatory and lesion-dependent leukocyte exacerbation [125,126,127,128] as well as enlarged astrocytosis after TSCI compared to TBI [57,125]. Neuroinflammation following central nervous system injury possesses a dual role [56,129]. As the innate and the adaptive immune response represent the foundation for central nervous system regeneration, they are also responsible for the neuroinflammation linked to accelerated neurodegeneration and chronic traumatic encephalopathy [55,57]. Therefore, injuries such as TSCI and TBI commonly result in chronic neuroinflammation, with astrocyte hyperactivation, pericyte persistence, extracellular matrix deposition and glial scar formation [55,57,58]. 

Although polytrauma, often accompanied by TBI and TSCI, is strongly associated with systemic inflammatory response syndrome (SIRS), despite advances in acute haemorrhage and coagulopathy management [130,131] surprisingly few studies focus on the systemic effects of TBI with additional extracranial injury [132].

SIRS is characterised by early innate hyperinflammation and delayed immunosuppression of the adaptive immune response [133], resulting in increased susceptibility to infection, sepsis and finally multiple organ failure [134,135]. TSCI and TBI patients were reported to suffer from systemic immune suppression, secondary immune deficiency syndrome, and hyperinflammation with chronic neuroinflammation and autoimmunity [122,123,136,137,138]. Along with systemic immune response dysregulation, the disruption of secondary lymphatic organ innervation following TSCI also results in immune paralysis (so-called secondary immunodeficiency) [139], which further increases susceptibility to infections [138,140].

The combined dysregulation of the autonomic nervous system and the systemic immune response following TSCI and TBI can have profound systemic effects on various peripheral organs [141,142] such as gastrointestinal dysfunction following the gut–brain axis [143,144]. Therefore, the gut microbial content of patients suffering from TSCI was correlated to immunological and functional outcome, proposing that gut microbiota may be involved in the increased infection susceptibility [145]. Overall, TSCI and TBI strongly modulate local and systemic immune responses, engaging neural, endocrine, paracrine, and cell–cell interactions.

### 3.6. Local Perfusion

During primary injury of TSCI, the direct insult to the spinal cord can cause severe haemorrhage [146], whereas the secondary injury, through the interruption of spinal cord vascular supply as a result of excessive bleeding and trauma-related neurogenic shock with hypovolemia and hemodynamic shock, can lead to increased spinal cord ischemia [27,147]. While larger vessels such as the anterior spinal artery commonly remain intact, rupture of smaller intramedullary vessels and capillaries causes extravasation of immune cells at the injury site [146] and endothelial injury-induced vasogenic oedema. These interactions cause additional pressure to the injured spinal tissues and, in addition to the haemorrhage-induced vasospasm, result in further disruption of the blood flow [27].

Similar to TSCI, the mechanical insult of the primary injury following TBI can result in either macroscopic injuries through direct bleeding after vessel injuries, or microscopic nerve tissue damage (such as diffuse axonal injury) and micro-vascular damage that causes inflammation and oedema, thus initiating the second injury phase. The secondary injury can result in alteration of the blood flow, ischemia, hypoxia, cerebral oedema and raised intracranial pressure [121,148]. The hypoperfusion from mass lesions or oedema is due to locally raised parenchymal pressures that decrease the local blood flow to levels below the normal cerebral perfusion pressure of ~55 mmHg. Furthermore, the local injury leads to a disruption of the normal vascular autoregulatory mechanisms, so the brain cannot compensate for the decreased perfusion.

In patients with TBI, modulations of systemic arterial pressure can cause alterations in cerebral blood flow leading to severe and potentially irreversible conditions such as hypoperfusion (brain ischemia) or hyperperfusion-induced oedema. Due to autoregulatory mechanisms, changes in cerebral blood volume or systemic arterial pressure can cause vasodilation or constriction of brain vessels. In spinal cord perfusion, similar autoregulatory mechanisms have been observed [149]. Therefore, low systemic arterial pressure caused by blood loss in severely injured patients is often associated with a drop in cerebral perfusion pressure. This triggers vasodilation of the cerebral blood vessels and subsequent increase in the cerebral blood volume. If the systemic arterial pressure drops below the lowest limit of the autoregulatory response, the cerebral perfusion pressure potentially reduces, resulting in brain ischemia. However, TBI studies show that affected patients particularly suffer from loss of autoregulatory capacity [150], decreased cerebrovascular reactivity [98], and blood–brain barrier leakage [151].

The understanding of TBI-TSCI interaction with regards to the local perfusion is limited. TBI activates the sympathetic nervous system with the release of endogenous catecholamines such as the vasopressor norepinephrine [152], which leads to reactive vasoconstriction of peripheral vessels in order to maintain an elevated mean of the systemic arterial pressure (neurogenic hypertension). This could positively affect the perfusion of the spinal cord in patients with additional TSCI. Exogenous norepinephrine was administered in acute TSCI patients in order to avoid hypotension and optimise spinal cord perfusion [153,154]. Elevated mean arterial blood pressure during the acute phase of TSCI was correlated with better long-term neurological recovery [149,155]. Therefore, post-TSCI blood pressure management with the goal of a mean arterial pressure over 85 mmHg has become the clinical standard [156,157]. Nevertheless, the reperfusion of ischemic tissue, which contains, e.g., cytokines, chemokines, reactive oxygen species and excitatory neuro-transmitters, can promote the ongoing inflammatory response during the secondary injury phase of TSCI, potentially resulting in hyperinflammation and tissue damage [155].

To date, detailed analysis of the spatiotemporal aspects of TSCI and TBI interaction regarding re-perfusion damage and maintenance of sufficient perfusion is missing; however, based on the available studies on isolated injuries interaction is likely, and warrants further analysis.

### 3.7. Endocrine Dysregulation

The endocrine system plays a pivotal role in the maintenance of whole-body homeostasis, especially after trauma and critical illness [158,159].

Following TBI, dysregulation of endocrine signalling cascades have been reported for various axes including insulin [160], pituitary dysfunction [161] with disruptions in the growth hormone (GH)/insulin-like growth factor 1 (IGF-1) axis [162], antidiuretic hormone (ADH, vasopressin) [163], the hypothalamic-pituitary-adrenal (HPA) axis [164], sexual hormones [164,165], thyroid-stimulating hormone (TSH) [166,167], alterations in leptin signalling [75,168], osteocalcin (OCN) [168] and lipocalin 2 [169,170]. Further analysis depicted a bidirectional interaction between the brain and the endocrine system in TBI [171,172].

Regarding acute TSCI, posttraumatic endocrine alterations are not that well characterised [173,174]. Following acute TSCI, dysregulation of the SNS and HPA axis occur, and are linked to post-TSCI immunosuppression [102,175,176]. The level of ACTH, cortisol and prolactin dysregulation may be affected by the level of injury [177]. Further, in acute and chronic TSCI, low levels of vitamin-D3 (Vit-D3) are observed [178]. In chronic TSCI, a disruption of the endocrine pathway regulation metabolism and skeletal health involving several adipokines, including Leptin, the SNS and Vitamin D3, has been described [173,177,179].

Other additional TBI injuries such as bone fractures also have a major impact on endocrine mechanisms. Following bone fracture and heterotopic ossification, alterations have been observed for OCN, insulin, [168], calcitriol (1-,25-Vit-D3) and calcidiol (25-VitD3) [180,181]. Consequent haemorrhage and anaemia can trigger elevated systemic erythropoietin (EPO) levels [182,183].

Endocrine signalling was also reported to influence TSCI healing [184]. Insulin [160], thyroid hormone T3 [185,186], GH/IGF-1 [187,188], Vit-D3, EPO [16,189] and gonadotropin-releasing hormone (GnRH) [190] were reported to positively effect TSCI regeneration and outcome. Disruption in ADH secretion inducing Syndrome of inappropriate antidiuretic hormone secretion (SIADH) was observed for TSCI and TBI [163], while HPA axis dysregulation [102,164] showed negative effects on TSCI healing, resulting in persistent complications.

Despite the lack of direct studies on endocrine interaction with TSCI–TBI, the available data shows that TSCI and TBI both have profound effects on the endocrine system and are likely to interact with each other through it. As a detailed understanding of the underlying mechanisms and their impact on TSCI regeneration still remains to be elucidated, further preclinical and observational studies are needed.

### 3.8. Post-Traumatic Metabolism 

The key function of metabolism is storage for a demand-adjusted supply of energy in order to provide resources for anabolic processes and eliminate the waste products of catabolism. Trauma causes severity-dependent changes in the tightly regulated process of metabolism, which can be divided into three phases, the early shock, catabolic, and anabolic phases. The initial shock phase is characterised by reduced systemic energy expenditure and systemic adaptions to maintain tissue perfusion and homeostasis within the first hours after trauma. An increase of pro-inflammatory mediators and catecholamine such as epinephrine initiate the catabolic phase, which is dedicated to the ‘fight for energy’ of the injured tissue. Here, the metabolic rate can increase up to ~20–25% or more [191,192]. This hypermetabolism is distinguished by an elevated body temperature and heart rate, high energy expenditure, peripheral stress-induced insulin resistance, and hyperglycaemia (plasma glucose levels >200 mg/dL) with extensive turnover in free fatty acids, followed by activation of gluconeogenesis, proteolysis and lipolysis [193,194]. If this metabolic stage stretches on too long due to the severity of the injury, adipose tissue, skin, muscle and other tissues can be destroyed. These alterations are further accompanied by additional structural and functional transformation, such as browning of the white adipose tissue (WAT) induced by prolonged adrenergic stress response [106]. Furthermore, brown adipose tissue (BAT) is activated upon trauma [105], resulting in elevated energy expenditure with increased glucose and fatty acid oxidation as well as insulin sensitivity [106]. With BAT activation, the final stage of posttraumatic metabolism begins, characterised by the accession of anabolic processes to recover the former systemic loss [193]. These stages of posttraumatic metabolism are commonly observed in patients suffering from TBI and TSCI [27,195,196,197,198]. Following TBI, the disruption of normal cellular and mitochondrial function [195,196,197,198,199] as well as, the increase of free radical production [196,198] have been described. The changes in glucose metabolism, especially hyperglycaemia, occur partially due to posttraumatic disturbed glucose transporter function (GLUT 1 and 3) as well as to the increased demand for energy, which is needed to restore the ionic balance and membrane potential [195,196,197,198,200], disrupted very early in the acute phase of TBI injury [201]. In the acute phase, the cell membrane is corrupted through the injury, which causes redistribution of ions and neurotransmitters, consequently altering the membrane potential. This in turn impairs mitochondrial function, initiates oxidative stress, increases free radical production and contributes to the changes in glucose metabolism [196,197,198,200] which have been linked to disturbed neural regeneration after TBI [199,201].

Along with the common posttraumatic metabolic events and alterations described for TBI, patients suffering from TSCI show aberrant occurrence of neurotransmitters, especially glutamate disruption due to vascular and tissue destruction [27]. Furthermore, injured muscular and skeletal tissue is rich in Ca^2+^ ions, and therefore affected by the ionic imbalance [202,203,204]. In detail, TSCI causes immediate and permanent unloading of the involved skeletal regions, with structural and metabolic effects triggering calciuria and hypercalcemia within 10 days and up to 1–6 months after injury [202]. Osteoblastic activity is further diminished, resulting in osteoporosis in the pelvis and the extremities affected by the spinal injury [202]. In addition to the profound metabolic changes which generally occur after trauma, a dominant effect of neuronal injury is the alteration of body composition and its long lasting metabolic consequences [204,205]. Hence, shortly after injury rapid and significant muscle atrophy, mainly below the level of injury, can be observed. The atrophy is partly trigged trough the reduction of hormones such as testosterone within a few weeks, and continues beyond the end of the first year [202,203,204,205]. The loss of metabolic active muscle mass results in the reduction of the basal metabolic rate and resting energy expenditure. This altered metabolism is reflected in the frequently observed obesity state of TSCI victims, resulting in further severe health consequences such as glucose intolerance, insulin resistance, hyperlipidemia, and diabetes [202,205]. The survival rate of TSCI is estimated to be 69–96%, further emphasising the importance of treating the affected metabolism [206].

Especially in patients with severe trauma, such as polytrauma with involvement of the central nervous system, the maintenance of metabolism represents a pivotal aspect of therapy [192]. Following TBI, insulin resistance, hyperglycaemia, ketones and distinct alterations in lipid profiles were commonly observed [160,196,207,208,209], while disturbed metabolic parameters were associated with a decreased neurological outcome in TBI [210]. As hyperglycaemia was shown in particular to negatively affect regeneration following TSCI and TBI in humans and in vivo [211,212,213,214], clinical trials were run to investigate the application of insulin to reduce the vital state of hyperglycaemia. Surprisingly the therapeutic approach with insulin resulted in controversial outcomes [160]. However, differential monitoring of glucose was shown to be effective for the limitation of secondary neuronal damage and the improvement of TBI outcomes [215,216]. Concomitant injuries in TSCI are therefore likely to contribute to inferior clinical outcomes through their disturbed metabolism. Although specific studies on the effects of co-occurring TSCI and TBI on posttraumatic metabolism remain limited, a more detailed understanding of this aspect will contribute to new nutritive and endocrine therapeutic strategies in the acutely injured, potentially overcoming negative interaction.

### 3.9. Circadian Rhythm

One essential mechanism that mediates the interaction of TBI, TSCI and peripheral organs is the circadian rhythm. It is controlled by a central pacemaker in the suprachiasmatic nucleus (SCN) of the hypothalamus that is synchronised with the light–dark cycle and regulates the intrinsic clocks in the peripheral organs [217]. Through its connection with other hypothalamic nuclei, the SCN controls the sympathetic nervous system as well as the hypothalamic-pituitary-adrenal axis in order to transmit the rhythm to different tissues throughout the body [218,219].

Disrupted circadian rhythm is a common symptom following TBI, and is reflected by the fact that up to 50% of these patients suffer from sleep disorders that are additionally characterised by aberrant expression patterns of clock genes in mononuclear blood cells [220,221,222]. Similar to TBI patients, those with cervical spinal cord injury commonly develop sleep disorders that are accompanied by circadian disruptions like dysregulated circadian rhythm and course of melatonin production [223,224], core body temperature [225], and aberrant expression patterns of clock genes in peripheral blood mononuclear cells [226]. Sleep abnormalities have been shown to negatively affect the outcome of TBI [227], and a recent study in mice shows involvement of circadian regulation in the neuroinflammation and blood–spinal cord barrier disruption following TSCI [228]; therefore, different kinds of chronotherapeutics have already been clinically tested. Blue-wavelength light therapy seemed to be helpful for patients with mild TBI or long-term fatigue following TBI [229,230]. The reduction of evening melatonin, often referred to as the ‘sleep hormone’, could lead to insomnia and was observed in patients suffering from spinal cord injury and TBI [224,231,232,233]. As melatonin acts as a neuroprotector and reduces oxidative stress as well as neuroinflammation, it might be a promising drug to treat sleep disorders following TSCI and TBI [226,234,235].

## 4. TBI, TSCI and MSCs

### 4.1. MSC Therapy in Post-Traumatic Neurological Disorders

Stem cells are multipotent cells with the ability to differentiate into various cell types, as well as, to renew themselves [236]. Mesenchymal stem cells (MSCs), hematopoietic stem cells (HSCs), brain-derived neural stem cells (NSCs), embryonic stem cells (ESCs), and induced pluripotent stem cells (iPSCs), which are reprogrammed from somatic cells, represent major stem cell sources previously investigated for traumatic brain injury, spinal cord injury, neurodegenerative disease and stroke therapy [84,237,238,239,240,241,242,243,244,245,246,247,248,249] (Table 1).

The distinct properties of MSCs places them among the most looked-for cell sources. Therapy development based on these cell types is currently rapidly evolving in regenerative medicine. The simplicity with which MSCs can be obtained from various sources as well as their low immunogenicity and immunomodulatory abilities makes them available for transplantation in both auto- and allogeneic systems. There have been 125 clinical trials using MSCs to treat neurological diseases registered to date [247], and they are the most studied cell population in registered trials for TSCI (Table 1). These cells also proliferate quickly and have a high level of multilineage differentiation. Furthermore, MSCs retain their regenerative potential even after cryopreservation and have “homing properties”, allowing them to migrate toward the lesion site [250]. MSCs are principally found in bone marrow (BM-MSC), adipose tissue (AD-MSC), and peripheral blood. They can be obtained from the umbilical cord (UC-MSC), the umbilical cord blood (CB-MSC), the urine, the amnion, and the placenta, [251,252]; however, BM-MSCs were the first to be discovered and are thus the most studied type of MSC. They frequently serve as the gold standard and were initially used in the majority of clinical trials [76,253].

BM-MSCs show the capacity for self-renewal and differentiate into muscle, bone, fat, cartilage and connective tissue in vivo [84]. Beyond that, their additional proposed ability to differentiate into diverse neural cell types led to their intensive application in clinical and preclinical trials of neurodegenerative diseases and trauma of the central nervous system [238,239,240,241,247,254,255,256,257,258,259]. Their primary function, however, is a modulating one in the case of TBI/TSCI. Therefore, BM-MSC treatment allows cell-specific differentiation [260] as well as the positive paracrine effect through cytokine release [254], resulting in the limitation of inflammatory secondary injury, promotion of neurogenesis and stimulation of neuronal progenitor cells maturing into neurons [261], all relevant for TSCI and TBI treatment.

Experimental results suggest a promising approach for clinical application of MSCs in TBI patients [245]. There have been a small number of clinical trials with MSC therapy for TBI to date. In TBI disorders, autologous BM-MSCs transplanted into the injured brain during cranial surgery had no negative effects [248]. The administration of autologous BM-MSCs via lumbar puncture to 97 patients with TBI in the subacute stage was also shown to be safe. Following MSC transplantation, approximately 40% of patients showed improved neurological function, in a non-controlled trial in patients with vegetative state after TBI [262]. Autologous BM-mononuclear cell delivery (containing BM-MSCs along with cells of hematopoietic and lymphocytic lineage) was observed to reduce neural cell loss, reduce neuroinflammation and improve clinical outcomes after TBI in adults and children in Phase I trials [263,264], while a phase I/IIa trial in adult TBI patients showed safety, however with only limited data on possible enhanced outcomes [265]. These studies may be limited by their design, as they used BM-mononuclear cells, which in addition to BM-MSCs also contain cells of hematopoietic and lymphocytic lineage [245,264,265]. For a brief summary of ongoing and completed clinical trials of stem-cell based therapies in TBI, we refer to a recent review [245] and Table 1.

In TSCI, BM-MSC treatment aims for optimal neuronal regeneration by limitation of the secondary injury and modulation of the local microenvironment in order to optimise healing cascades and axonal regeneration as well as by targeting differentiation of cells to restore spinal cord function [17,19,266]. MSC transplantation after TSCI has shown promising results in some preclinical studies [258,267,268,269]. These results were translatable to the chronic phase of the injury in mice [259,270]. Single MSC application was demonstrated to be safe but had little therapeutic outcome in a phase three clinical trial [271], which was supported by former and recent systematic reviews summarising clinical and preclinical evidence [19,272,273]. There are currently several trials in progress and their completion will deliver the required data on the efficacy of MSC therapies after TSCI [84,274]. For a brief summary of stem-cell based therapies in TSCI, we refer to Table 1 and other recent reviews [19,246,272,275]. Overall, MSC therapy improves the microenvironment of the injury site, improves nutritional support, modulates the inflammatory response, and reduces blood–brain and blood–spinal cord barrier leakage, all of which help with TSCI healing [274]. Single cell types, on the other hand, have limited proliferation, therapeutic efficacy, homing ability, and survival [84]. To improve TSCI regeneration, cell-based therapies may be crucial; however, they require further detailed investigation and clinical trials.

### 4.2. The Potential Effect of TBI on MSC-Based TSCI Treatment

Preclinically observed BM-MSC-induced improvements in TSCI outcome remain far from a restitutio ad integrum [272,276]. Therefore, this review seeks to raise awareness about the impact of additional trauma, such as frequently concomitant TBI, on MSC treatment.

**Figure 5:**
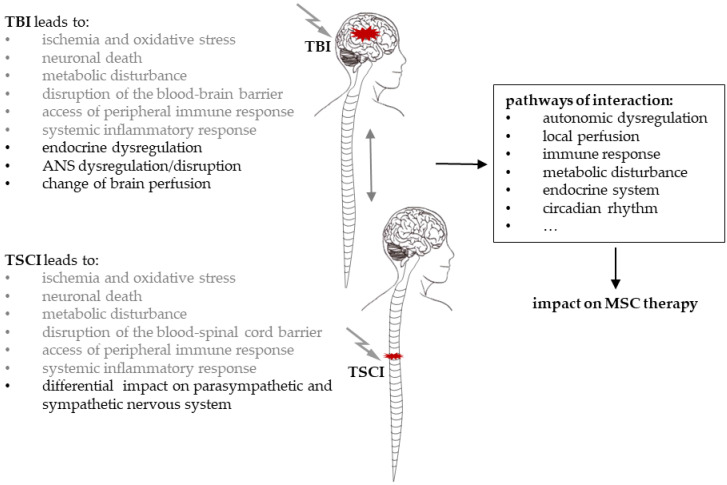
Interactive response of TSCI and TBI potentially effecting MSC treatment. Although TSCI and TBI feature common local and systemic effects (grey), several responses to spinal cord and brain injury are unique (black). Therefore, different pathways of interaction can be identified and should be considered in order to optimise mesenchymal stem cell (MSC) treatment in patients suffering from TSCI with concomitant TBI. Abbreviations: ANS = autonomous nervous system, TBI = traumatic brain injury, TSCI = traumatic spinal cord injury.

TSCI and TBI are proposed to interact in a bidirectional manner, suggested by the negative effect of TSCI on cognitive function in patients suffering from TBI, the impact of TBI on sensorimotor outcome in patients with TSCI, and the clinical observation of enhanced ossification following TSCI and TBI, underlining the impact on MSC mediated healing cascades. Therefore, TSCI-TBI interactions on the local and systemic levels still have to be characterised and considered upon the application of MSC therapy. These environmental changes are caused by the alterations of the autonomic and peripheral nervous system, the inflammatory response, local perfusion, endocrine homeostasis and circadian rhythm (Figure 5), potentially causing crucial effects on MSC biology.

Successful MSC-based therapy in TSCI therefore depends on: (I) stem cell survival, viability and secretomic capacity; (II) cell roaming to the injury side and subsequent differentiation; (III) limitation of secondary injury; and (IV) optimised neural regeneration [19,277,278].

(I) Stem cell survival: Following severe TSCI and implantation of stem cells in the acute phase, survival of these cells in TSCI was markedly reduced [84,279]. Regarding concomitant injuries there are currently no data, but further reduced cell survival due to compromised local perfusion and dysregulated systemic and local energy metabolism such as hyperglycaemia is very likely. Concerning secrotomic capacity, it has been shown that inflammatory cytokines inhibit the proangiogenic capacity of the soluble component of the MSC secretome [280], and that the secretomic capacity of BM-MSCs is crucial for the promotion of neuronal survival after TSCI [281].
(II) Cell roaming and differentiation: Interestingly, TSCI and TBI exert differential stimulatory effects on neuronal stem cell niches in the brain, with potential effects on cell recruitment [282]. Regarding MSC chemotaxis to the injured spinal cord, CGRP—which is strongly regulated after TBI—showed a key role in vitro and ex vivo [283]. Antibody blockading of interleukin 6, which is strongly upregulated after concomitant injuries and severe infection in mice with TSCI and MSC treatment, improved MSC survival and locomotor function. The GH/IGF-1 axis, including the parathyroid hormone (PTH) and VitD3, are crucially involved in chondrogenic and osteogenic MSC differentiation as well as MSC-mediated angiogenesis [284,285,286], while local hypoxia enhances MSC proliferation in vitro [287]. As both TSCI and TBI cause heterotopic ossification, positive effects on MSC proliferation as well as negative osteogenic effects on MSC differentiation after severe trauma could be limiting aspects that require further research.
(III) Limiting secondary injury: As severe autonomic dysregulation affects whole-system energy metabolism via distinct effects on gastrointestinal function, glucose and lipid distribution, metabolism, and browning of adipose tissue, direct and indirect effects on MSC treatment in TSCI are very likely. Regarding limiting secondary injury with transplanted MSC, they are directly affected by adrenergic signalling; stimulatory and inhibitory proliferative effects have been described [288,289,290], while increased survival under challenging conditions such as hyperglycaemia and oxidative stress [110,291] were also observed. Overall, the data on SNS impact on MSCs in trauma is limited, while PNS effects on MSCs are little understood at present. Regarding circadian rhythm, melatonin has been shown to be a relevant factor in MSC treatment of TSCI in vivo [292,293]. In MSCs, and derived cell types a significant number of genes show circadian expression, regulating their differentiation and activity [294,295,296,297]. Melatonin preconditioning of these cells could improve their regenerative potential [298,299,300]. As TSCI and TBI both negatively affect circadian rhythm as well as circadian-mediated inflammatory and healing cascades [227,233,301,302], chronotherapeutic aspects in MSC therapy for TSCI should be considered.
(IV) Optimised neural healing: MSC-based therapy was reported to positively affect neural healing in TSCI, specifically through enhanced axonal regeneration and reduced glial scarring via the paracrine effects of secreted cytokines, exosomes, and local mediation of inflammatory response [274]. Specifically, modulation of the local inflammatory micromilieu by an MSC-mediated shift in macrophage polarisation towards M2 [303], as well as an exosome-induced reduction in astrocyte-mediated posttraumatic neurodegradation [304] was observed. As previously stated, TBI induces relevant inflammatory peripheral modulation [94], systemic and peripheral inflammation [305] and disturbed microbiota [89], and has been linked to enhanced bone healing by M2 polarisation in clavicle fractures [306]. In association with TSCI, these effects have not been addressed, although some impact of TBI on MSC-mediated regeneration following TSCI can be expected.

Overall, MSCs have a regulatory phenotype; they respond quickly to environmental signals that control their biological and secretorial activity [84,280]. The varied impacts of concurrent TBI and TSCI on MSCs are not fully explored; however, the available data suggest that there is an easily discernible association between the existence of additional trauma such as TBI and the efficacy of MSC-based therapy in TSCI, warranting further research attention.

## 5. Outlook: Brain as Saviour?

TSCI and TBI both show distinct effects on posttraumatic pathophysiology, with trauma-dependent differences. Although isolated TSCI and TBI have been intensively studied in basic translational and clinical research, understanding of the pathophysiological mechanisms of the frequently co-occurring injuries remains limited. Stem cell-based therapies, especially MSC treatment for patients suffering from TSCI or TBI remain a promising regenerative therapy towards restitutio ad integrum, even if it has until now provided only mild benefits [19]. One central limitation is the complex process of neural regeneration with crucial spatiotemporal aspects, which is not yet fully understood [307]. A second limitation is the expected complex interaction of TSCI and TBI pathophysiology.

As TSCI and TBI negatively affect each other, their underlying pathophysiology might reveal crucial insights on systemic posttraumatic interactions and local spatiotemporal aspects of neural regeneration. Therefore, we reviewed the current literature on systemic effects and possible interactions of both injuries. In the clinical setting, the additive trauma of TSCI and TBI causes reduced neurologic recovery as well as rising morbidity and mortality. Both injuries represent primary local neurotrauma with subsequent somatotopic effects on neurological function (Section 3.1), also resulting in reduced neurological function in areas that were not primarily injured [308,309,310,311] (Section 3.2). In summary, TSCI and TBI show profound systemic pathophysiological effects, particularly regarding immune response, autonomous regulation, perfusion, circadian rhythm and posttraumatic metabolism (Section 3).

Especially in the early posttraumatic phase of TSCI and TBI, autonomic dysregulation with aberrant SNS activity (Section 3.3) contributes to altered cerebral and spinal-cord perfusion (Section 3.6), immune modulation (Section 3.2 and Section 3.5), disrupted endocrine signalling, metabolism and energy-wasting (Section 3.7 and Section 3.8) as well as dysregulation of the circadian rhythm (Section 3.9). Regarding cell-based therapies (Section 4.1), the effects of these alterations and the SNS itself have to be considered as, e.g., adrenergic signalling strongly influences MSC differentiation [111] (Section 4.2). The role of SNS pathophysiology in TSCI and TBI has been researched for decades [29,312,313,314,315,316,317]; however, in recent years the role of SNS disruption in posttraumatic pathophysiology has come into focus [83,102,318], and the SNS is the target of new therapeutic interventions in TSCI and TBI [83,319,320]. However, comparative studies on the interaction of SNS in TBI and co-occurring TSCI are still missing.

Questions concerning TSCI–TBI interaction continue to evolve. What happens after disruption of the blood–spinal cord barrier following TSCI, subsequent loss of cerebrospinal fluid (CSF) to the surrounding tissue, and consequent local inflammatory and systemic effects, particularly in the context of TBI? What are the distant effects after sensitization of the adaptive immune system to the central nervous system and the loss of its immunological privilege? How do TSCI and TBI together impact posttraumatic immunodeficiency, metabolic disturbance, energy-flux and thermogenesis? Is there a race for glucose between TSCI and TBI? How do they interact with further injuries, especially with respect to perfusion and blood pressure? Are the systemic effects of concomitant injuries underestimated in MSC therapies to date? Can successive experiments on TBI–TSCI interaction unravel hitherto unknown pathophysiological pathways, suitable for new targeted therapies? As assumptions concerning possible interactions are speculative, systemic approaches with respect to the systemic pathophysiology of these traumata are urgently needed in order to understand TSCI–TBI interaction on the local as well as the systemic level. Advancements in ‘-omics’ aim for spatial and temporal resolution, leading to new perspectives on local healing cascades [321,322].

For future attempts in spinal cord regeneration, trauma-dependent systemic pathophysiology has to be considered for optimised survival of regenerative constructs and targeted protection against secondary injuries. As the temporal and spatial aspects of neural regeneration are crucial and potentially disturbed by systemic influences, targeted environmentally-triggered modulation of healing (e.g., by complex, logic based, scaffold cytokine release systems, combined cell therapies [243,323,324,325]) represents an evolving concept that in synergy with optimised surgical, intensive and rehabilitative care might overcome today’s therapeutic limitations. Therefore, exploring the complex interaction of TSCI and TBI and their impact on stem cell therapies will provide a better understanding of ‘the brain’s’ impact on TSCI pathophysiology and regenerative therapies, potentially rescuing spinal cord function in the future.

**Table 1:** Overview of different cell types used in TSCI and TBI treatment studies and recent reviews regarding those cell therapy strategies in TSCI and TBI. Different cell types display specific favourable and disadvantageous characteristics for therapy in TBI and TSCI, as summarized in the second and third column. For TSCI, numerous registered clinical trials were identified, of which nearly ½ were completed and 1/3 have published results. For TBI there were fewer trials identified, with only three published results. While some trials have led to more than one paper publishing results, other papers include results from more than one registered trial. Compared to some published reviews also including non-registered trials [326], our numbers of trials and publications are clearly lower. Under registration of clinical trials may be a relevant aspect, beyond this issue [327]. Nevertheless, considerable data on MSC therapy in TSCI is available, displaying safety [275], while optimal application and demonstration of relevant treatment effects warrant further studies.

	Proposed Advantages	Proposed Limitations	Clinical Trials TSCI (Total/Completed/Published)	Clinical Trials TBI (Total/Completed/Published)	Recent Reviews Cell Therapy And TSCI	Recent Reviews Cell Therapy and TBI
**Cell Therapies**						
**Omnipotent Cells**						
Embryonal stem cells (ESCs)	- omnipotency, possibility of in vitro pre-differentiation to desired cell subset (e.g., neuronal or oligodendral precursor cells) - direct neuronal replacement - direct glial replacement - replacement of endothelial cells - secretomic activity	- ethical concerns - immunogenity - tumourigenesis			Systematic: [275,328,329] Narrative: [84,330,331,332,333,334]	Narrative: [245,264]
Induced pluripotent stem-cells (iPSCs)	- autologous transplantation possible with reduced immunogenity - lack the ethical concerns of ESCs - omnipotency, possibility of in vitro pre-differentiation to desired cell subset (e.g., neuronal or oligodendral precursor cells) - direct neuronal replacement - direct glial replacement - replacement of endothelial cells - secretomic activity	- tumourigenesis				
**Multipotent cells & differentiated cells**			73/36/25	14/6/3		
**Cells of (Neuro-) Ectodermal Lineage**			13/9/7	0/0/0		
Neural stem cells (NSCs)	- direct neuronal replacement - neuronal, oligodendral and astrocytic differentiation possible - potential of remyelination - modulation of microenvironment - promotion of oligodendrocyte survival	- ESC or iPSC as source mostly needed (one MSC based therapy reported (NCT02326662)) - immunosuppression regiments in allogenous strategies	6/5/5 [335,336,337,338]		[339]	
Neural precursor cells (NPCs)	- direct neuronal replacement - secretomic activity (e.g., trophic factors) - modulation of microenvironment		1/0/0			
Schwann cells (SCs)	- harvestable from peripheral nerves - promotion of local substrate to faciliate axonal growth - remyelinisation (direct and indirect)	- no neuronal differentiation	2/2/2 [340,341]			
Olfactory ensheathing cells (OECs)	- minimal inasive harvesting from the nasal mucosa or olfactory bulb - promotion of local cellular substrate to faciliate axonal growth - remyelinisation - local immunomodulation - guidance of axonal regeneration - roaming to the injury side	- no neuronal differentiation - some (not NCT registered) studies with embryonal cells - limited cell survival and function	2/0/0		[326]	
oligodendrocyte precursor cell (OPC)	- secretomic activity (e.g., trophic factors)- remyelinisation- local immunomodulation- stimulation of angiogenesis	- ESC or iPSC as source mostly needed- immunosuppression regiments in allogenous strategies	2/2/0			
**Cells of Mesodermal Lineage**			60/27/18	14/6/3		
Bone marrow derived cells/aspirate (BMCs)	- minimal invasive autologous harvesting possible - direct intraoperative processing and application - containing haematopoietic and mesenchymal stem cells and endothelial progenitor cells - immunomodulation - guidance of axonal regeneration	- low survival rate in CNS - donor variability in allogenic products - heterogenic cell populations - ectopic migration	1/1/1 [342]			
Bone marrow derived stem cells (BM-SCs)	- long experience in harvesting and safe systemic application due to leukaemia treatment - minimal invasive autologous harvesting possible - containing haematopoietic and mesenchymal stem cells - immunomodulation - low immunogenicity		6/3/1 [343]	2/0/0	[344]	
Bone marrow derived mononuclear cells (BM-MNCs)	- minimal invasive autologous harvesting possible - containing haematopoietic and mesenchymal stem cells - immunomodulation - preservation of blood–brain barrier		7/0/0 2 × withdrawn	5/4/3 [265,345,346,347]	[348]	[264]
Bone marrow derived mesenchymal stem-cells (BM-MSCs)	- minimal invasive autologous harvesting possible - low immunogenicity - migration to damaged tissue - no ethical concerns - neuronal trans-differentiation - favourable secretome, production of favourable microvesicles - neurotrophic signalling - promotion of angiogenesis - immunomodulation - mitrochondrial transfer - inhibition of gliosis - prevention of apoptosis	- role of in vivo neuronal trans-differentiation unclear - low survival rate in CNS - donor variability in allogenic products - ectopic migation - tumourigenicity still discussed	17/10/9 1 × suspended [271,349,350,351,352,353,354,355,356]	1/1/0 1 × interim data published [357]	[19,268,272,273,358]	[359,360,361,362]
Adipose tissue derived mesenchymal stem cells (AD-MSCs)	- autologous harvesting possible, ubiquitous availability - faster proliferation than BM-MSC - migration to damaged tissue - no ethical concerns - see BM-MSCs		14/4/2 [363,364] 1× publication of interim data [365] 4x individual patient expand access	3/0/0 1 × withdrawn		
Umbilical cord-derived mesenchymal stem cells (UC-MSCs)	- non-invasive harvesting - higher proliferation and differentiation capacities than other MSC sources - migration to damaged tissue - see BM-MSCs	- role of in vivo neuronal trans-differentiation unclear - low survival rate in CNS - ectopic migration - autologous approach logistically difficult	11/5/2 1×withdrawn [366,367]	2/0/0 1× withdrawn		
Umbilical cord derived cells (UC-MNCs & UC-MSCs)			3/2/2 [368]			
further and undefined MSCs			3/2/1 [369]	1/1/0		
Macrophages	- autologous therapy possible - favourable local immunomodulation	- pulmonary embolism	1/0/1 [370] (suspended)			
**Sum**			73/36/25	14/6/3		

## Data Availability

The the data is available on reasonable request from the authors.
